# A predictive PC-SAFT EOS based on COSMO for pharmaceutical compounds

**DOI:** 10.1038/s41598-021-85942-8

**Published:** 2021-03-19

**Authors:** Samane Zarei Mahmoudabadi, Gholamreza Pazuki

**Affiliations:** Department of Chemical Engineering, Amirkabir University of Technology (Tehran Polytechnic), Tehran, Iran

**Keywords:** Chemical engineering, Biotechnology

## Abstract

The present study was conducted to develop a predictive type of PC-SAFT EOS by incorporating the COSMO computations. With the proposed model, the physical adjustable inputs to PC-SAFT EOS were determined from the suggested correlations with dependency to COSMO computation results. Afterwards, we tested the reliability of the proposed predictive PC-SAFT EOS by modeling the solubility data of certain pharmaceutical compounds in pure and mixed solvents and their octanol/water partition coefficients. The obtained RMSE based on logarithmic scale for the predictive PC-SAFT EOS was 1.435 for all of the solubility calculations. The reported values (1.435) had a lower value than RMSE for COSMO-SAC model (4.385), which is the same as that for RMSE for COSMO-RS model (1.412). The standard RMSE for octanol/water partition coefficient of the investigated pharmaceutical compounds was estimated to be 1.515.

## Introduction

Computation of phase equilibria and thermodynamic properties is believed to be one of the significant objects in thermodynamic investigations, which directly relates to molecular structure, functional group, and chemistry of molecule. Quantum chemistry, as an approach to calculating the mechanical properties of molecules, has been extensively applied in thermodynamic computations. Quantum chemistry computation based on *ab* initio and density functional methods has been widely utilized for estimating the heat of formation and reaction, heat capacities, and molecular structure and geometry^1^. The Conductor-like Screening Model (COSMO), a continuum solvation model based on quantum chemistry, was firstly introduced by Klamt and Schuurmann^2^. The remarkable advantages of COSMO calculation in predicting physical and chemical properties were a motivation to expand its applications by employing different software. Several software packages, such as GAMESS^3^ and Dmol^3^^4^, were designed for COSMO-based quantum chemistry calculations. Consequently, some thermodynamic models have been developed based on COSMO calculations. The activity coefficient models based on COSMO, such as COSMO-RS^5–7^ and COSMO-SAC^8^, have been known as successful tools in thermodynamic scope. Studies by Tung et al.^9^, Zhou et al.^10^, Paese et al.^11^, Xavier et al.^12^, Bouillot et al.^13^, Shu and Lin^14^, Buggert et al.^15^, and Mahmoudabadi and Pazuki^16^ are some examples. Despite the remarkable characteristic of COSMO-based model, the vapor–liquid equilibria in COSMO-based model are computed by ideal gas assumption for vapor phase. Meanwhile, compressibility factor, viscosity, and different chemical and physical properties for various phases could be obtained via the equations derived from equations of state. The above-mentioned discussions have encouraged scientists to explore the remarkable properties of quantum chemistry calculations in combination with other equations of state in two forms: (i) combination with other equations of state (EOS) in the form of contribution in order to increase accuracy and (ii) combination with other equations of state in order to perform a predictive model.

In method (i), several researchers have examined adding quantum chemistry models, for instance, COSMO-RS^5–7^ and COSMO-SAC^8^, to other equations of state. Lee and Lin^17^ considered a combination of COSMO-SAC and Peng-Robinson (PR) for thermodynamic modeling. Pereira et al.^18^ examined the COSMO-RS model in selecting an association scheme and molecular models for soft-SAFT EOS in the ionic liquids system. Cai et al.^19^ investigated the PR + COSMO-SAC model for solid solubilities in supercritical carbon dioxide.

In method (ii), several studies have considered quantum chemistry computations combined with other commonly used equations of state toward a new predictive model for thermodynamic computations. In this method, the quantum computations are not directly incorporated in equations of state. The popularly applied equations of state have some adjustable parameters with physical meanings, which are tuned by experimental data. However, in this method, the adjustable physical parameters of EOS are determined based on quantum chemistry computations. There are certain studies in this regard as reported in Table 1. Milocco et al.^20^ considered two alternative methods based on COSMO calculations for estimating the PHSCT EOS parameters in order to predict vapor–liquid equilibria (VLE) behavior for refrigerants: (i) PHSCT EOS parameters were fitted by the computed activity coefficients from COSMO-RS model and (ii) PHSCT EOS parameters were obtained directly from quantum calculations. Cassens et al.^21^ utilized electrostatic properties of pharmaceutical compounds to identify Perturbed-Chain Polar Statistical-Associating Fluid Theory (PCP-SAFT EOS) parameters. The PCP-SAFT pure-component size and shape parameters of solutes were obtained from quantum chemistry by considering available size and shape parameters for PCP-SAFT EOS and introducing some relations between dispersion energy and reduced dipole moment to size and shape parameters. Ferrando et al.^22^ proposed a new methodology based on Monte Carlo simulations in order to determine the PPC-SAFT EOS-associated scheme and parameters by focusing on 1-alkanol molecules. Van Nhu et al.^23^ computed PCP-SAFT EOS parameters for 67 compounds using quantum chemical *ab* initio and density functional theory methods. Van Nhu et al.^23^ induced a relationship between quantum calculations and pure parameters for PCP-SAFT EOS. They tuned PCP-SAFT EOS parameters with vapor pressure and density data and changed atomic radii inputs to COSMO calculations so that it match with the obtained parameters of PCP-SAFT EOS. Singh et al.^24^ employed the *Gaussian03* software package in performing quantum results. They utilized different theories for obtaining the dispersion energy, the dipole moment, the quadrupole moment, and the dipole–dipole dispersion coefficient. Leonhard et al.^25,26^ proposed a novel method for computing PCP-SAFT parameters and introduced a combining rule based on quantum chemistry. They calculated the dispersion energy, the dipole moment, the quadrupole moment, and the dipole–dipole dispersion coefficient with quantum calculations and tuned the remaining parameters by experimental data.

**Table 1:** A list of recent studies on performing the predictive EOS based on quantum chemistry.

Study	Equation of state	Quantum chemistry model
Milocco et al.^20^	PHSCT EOS	COSMO
Cassens et al.^21^	PCP-SAFT EOS	COSMO
Ferrando et al.^22^	PPC-SAFT EOS	Mont Carlo
Van Nhu et al.^23^	PCP-SAFT EOS	*ab* initio, density functional theory and COSMO
Singh et al.^24^	PCP-SAFT EOS	*Gaussian03*
Leonhard et al.^25,26^	PCP-SAFT EOS	Density functional theory
This Work	PC-SAFT EOS	COSMO

According to the literature review, the quantum chemistry was mostly manipulated in PC-SAFT type EOS to perform a predictive model. However, a few studies considered original PC-SAFT EOS and constructed a predictive PC-SAFT EOS using quantum chemistry. Based on the methods implemented for performing predictive EOS, the majority of the studies have relied on the complicated equations and methods to estimate the parameters. Some others utilized the previously tuned parameters to define their equations. There is no study in which COSMO results are directly utilized for setting up their EOS. In this work, the adjustable parameters for PC-SAFT EOS could directly calculate through simple equations with decency to obtain the results based on COSMO calculations. The current study aimed to make a predictive PC-SAFT EOS by incorporating COSMO calculations from the Dmol^3^ module in Material studios 2017 with the discussed characteristics. The newly performed predictive PC-SAFT EOS was applied for estimating pharmaceutical compound solubilities in various pure and binary solvents. The octanol/water partition coefficients of the considered pharmaceutical compounds were also obtained with the proposed predictive PC-SAFT EOS. They were then compared to the experimental data. Through this modification to PC-SAFT EOS, the domains of applicability of COSMO computations would expand and the adjustable parameters of PC-SAFT EOS could be estimated.

## Thoery

### COSMO file

In quantum chemistry, molecular properties are determined via several electron time-independent equations^3^:

1 $${\mathbf{{\rm H}}}\Psi = \left[ {{\mathbf{T + U + V}}} \right]\psi = \left[ {\sum\limits_{i}^{n} { - \frac{{\overset{\lower0.5em\hbox{$\smash{\scriptscriptstyle\rightharpoonup}$}} {h} ^{2} }}{{2m}}\nabla _{i}^{2} + \sum\limits_{{i < j}} {U\left( {\overset{\lower0.5em\hbox{$\smash{\scriptscriptstyle\rightharpoonup}$}} {r} _{i} ,\overset{\lower0.5em\hbox{$\smash{\scriptscriptstyle\rightharpoonup}$}} {r} _{j} } \right)} + \sum\limits_{i}^{N} V \left( {\overset{\lower0.5em\hbox{$\smash{\scriptscriptstyle\rightharpoonup}$}} {r} _{i} } \right)} } \right]\psi = {\mathbf{E}}\Psi$$

where ${\mathbf{\rm H}}$ is electronic molecular Hamiltonian and ${\mathbf{T}}$,${\mathbf{U}}$, and ${\mathbf{V}}$ respectively imply electron, its interaction with other electrons, and external field. In COSMO, the solute molecule represents a cavity within the dielectric continuum of solvent with specific permittivity. The charge distribution of the solute polarizes the dielectric medium of the solvent. The dielectric medium responds to charge distribution by the generation of screening charges on the cavity surface. In COSMO calculations, the solute molecule assumes to have infinite permittivity, in which the screening charges locate on its molecular surface. To proceed with the quantum mechanics calculation, one needs to provide the electrostatic charge and its location in space. The locations of the surface charges are determined via some cavity and surface construction algorithms. The screening charges are determined with the boundary condition of vanishing electrostatic potential on the cavity surface owing to the surrounding perfect conductor. COSMO does not require a solution to the rather complicated boundary conditions for a dielectric for obtaining screening charges. Instead, the screening charges are calculated based on the density functional theory^4^.

In this study, 35 pharmaceutical compounds and 15 solvents were considered for solubility calculations. Performing the predictive PC-SAFT EOS, we examined 26 non-associating organic compounds, such as alkanes and alkenes, and 13 associating organic compounds, alkanols for instance. For all of the 80 surveyed compounds, the COSMO files from the Dmol^3^ module in Material studios 2017 software were generated. According to Lin and Sandler ^8^, GGA (VWN-BP) is considered as a density function, and the accuracy of computation is set by quality fine. In electronic options, multipolar expansion is selected octupole. The calculations are run at four parallel cores. The other options are set to the default values of DMol^3^.

**Figure 1:**
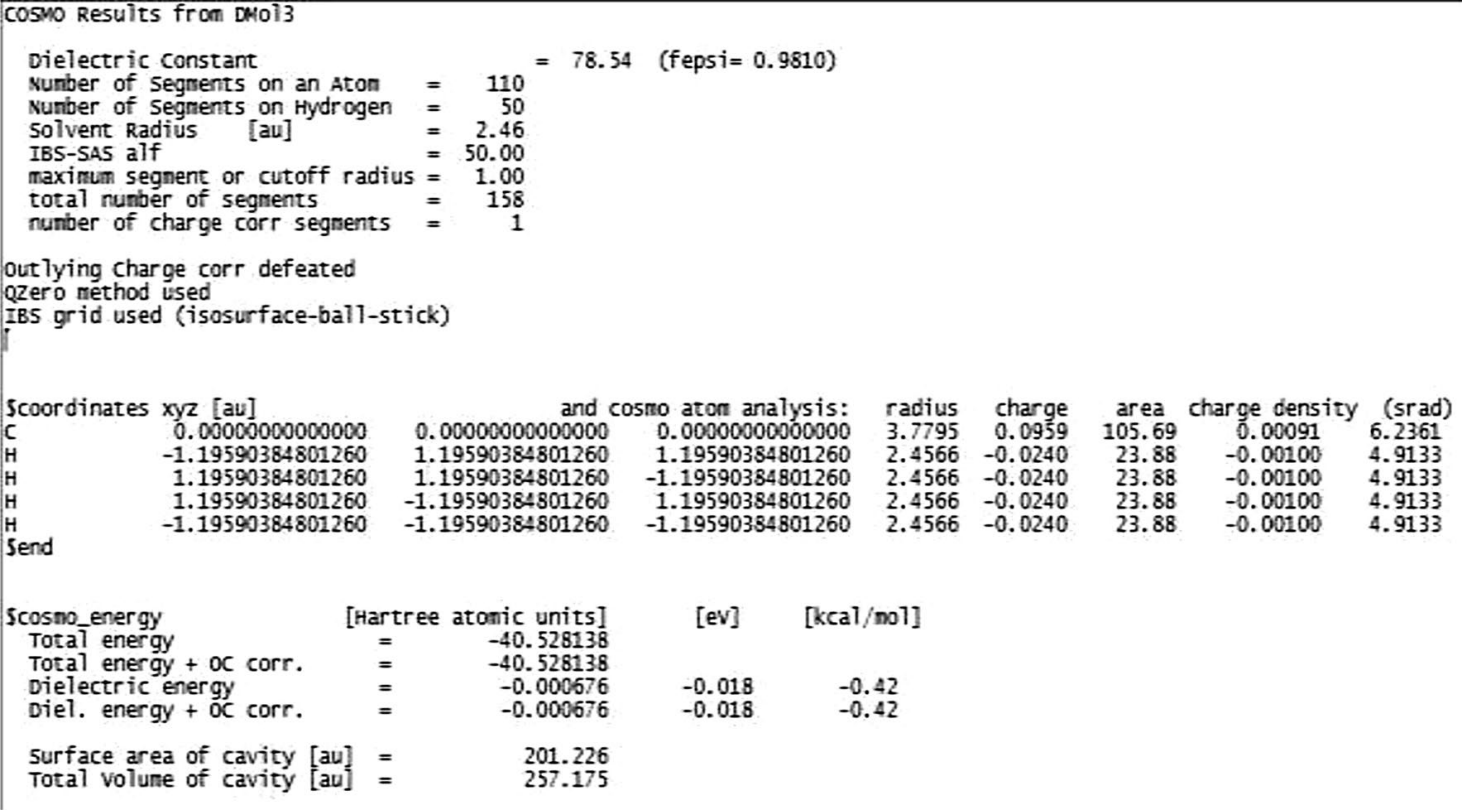
A schematic view of the COSMO file for methane.

Figure 1 displays a layout of the COSMO file obtained for methane. To perform a predictive PC-SAFT EOS, the surface area of the cavity (*A*) and the total volume of the cavity (*V*) were the desirable properties gathered for all of the 80 examined compounds. Prior to using the surface area of the cavity and total volume of the cavity, a unit conversion from Bohr to angstrom is required (1 Bohr = 0.52918 Angstrom).

### PC-SAFT EOS

Perturbed-Chain Statistical-Associating Fluid Theory (PC-SAFT) was firstly developed by Gross and Sadowski^27^. In PC-SAFT EOS, the perturbation theory for dispersion forces and hydrogen bonding were incorporated into a hard-chain reference fluid through the following equation:

2 $$a^{PC - SAFT} = a^{hc} + a^{disp} + a^{assoc}$$

in which the residual molar Helmholtz energy of the PC-SAFT (*a*^PC-SAFT^) is obtained by the Helmholtz energy contributions from the reference system, hard chain (*a*^hc^), dispersion force (*a*^disp^), and hydrogen bonding (*a*^*assoc*^) as follows^27,28^:

3 $$a^{HC} = ma^{HS} - \sum\limits_{i} {x_{i} (m_{i} - 1)\ln \left( {g_{ii}^{HS} \left( {\sigma_{ii} } \right)} \right)}$$

4 $$a^{disp} = - 2\pi \rho I_{1} (\eta ,\overline{m})\overline{{m^{2} \varepsilon \sigma^{3} }} - \pi \rho \overline{m}C_{1} I_{2} (\eta ,\overline{m})\overline{{m^{2} \varepsilon^{2} \sigma^{3} }}$$

5 $$a^{assoc} = \sum\limits_{i} {x_{i} \left[ {\sum\limits_{{A_{i} }} {\left( {\ln X^{{A_{i} }} - \frac{{X^{{A_{i} }} }}{2}} \right) + \frac{1}{2}M_{i} } } \right]}$$

where $X^{{A_{i} }}$ is not bonding mole fraction of component *i* at site *A*. $g_{ii}^{HS}$ shows the interaction between two spheres of component *i* in its mixture with hard-sphere contacts. *M* is the number of associated sites. The details of formulations of the above-mentioned equations were presented for non-associating contributions in Gross and Sadowski^27^ and for associating contributions in Huang and Radosz^29^.

The total five parameters of PC-SAFT EOS for each molecule, segment number *m*, segment diameter $\sigma$, dispersion energy $\varepsilon$, association volume $\kappa^{AB}$, and association energy $\varepsilon^{AB}$ are usually defined according to experimental data.

In this study, the solubility of pharmaceutical compounds in different solvents were desirable properties determined by knowing the activity coefficient of pharmaceutical compounds ($\gamma_{i}$) in its solutions. It was obtained as follows:

6 $$\gamma_{i} = \frac{{\hat{\varphi }_{i} }}{{\varphi_{i,pure} }}$$

In the above-mentioned equation, the $\hat{\varphi }_{i}$ and $\varphi_{i,pure}$ are fugacity coefficients for component *i* in the mixture and pure states at the same system temperature and pressure, respectively. The fugacity coefficient for component *k* ($\hat{\varphi }_{k}$) and compressibility factor (*z*) were estimated using the PC-SAFT EOS compute as follows:

7 $$\ln \hat{\varphi }_{k} = a^{PC - SAFT} + (z - 1) + \left( {\frac{{\partial a^{PC - SAFT} }}{{\partial x_{k} }}} \right)_{{T,V,x_{i \ne k} }} - \sum\limits_{j = 1}^{N} {\left[ {x_{j} \left( {\frac{{\partial a^{PC - SAFT} }}{{\partial x_{k} }}} \right)_{{T,V,x_{i \ne j} }} } \right]} - \ln z$$

8 $$z = 1 + \rho \left( {\frac{{\partial a^{PC - SAFT} }}{\partial \rho }} \right)_{{T,x_{i} }}$$

where T, $\rho$, and x respectively represent temperature, molar density, and mole fraction.

In order to obtain accurate results for the mixtures, the binary interaction parameter (*k*_*ij*_) is defined for correcting the segment-segment interactions of unlike chains and calculated with the combining rule below:

9 $$\varepsilon_{ij} = \sqrt {\varepsilon_{i} \varepsilon_{j} } (1 - k_{ij} )$$

The addition of an extra binary interaction parameter (*k*_*ij*_) increases the flexibility of the PC-SAFT EOS along with accurate modeling of more complicated systems.

### Solid/liquid equilibria

In solid–liquid equilibria, the solid solubility in the liquid phase is calculated according to the following expression ^30^:

10 $$\ln x_{i} = \frac{{\Delta H_{m} }}{R}\left( {\frac{1}{{T_{m} }} - \frac{1}{T}} \right) - \ln \gamma_{i}$$

where $x_{i}$ and $\gamma_{i}$ represent the solubility and activity coefficient of compound *i*. In this study, the activity coefficient of compound *i* ($\gamma_{i}$) was determined via Eq. 6. Since the activity coefficient depends on solubility in mole fraction ($x_{i}$), solubility must be determined from the iterations with Eq. 10. In the equation above, $\Delta H_{m}$ and $T_{m}$ represent fusion enthalpy and melting point temperature, respectively, and their values are presented in supplementary materials (Table S1).

### Octanol/water partition coefficient

Once pharmaceutical compounds are dissolved in two immiscible liquid phases containing octanol and water, the components will be distributed between two phases. The distribution of component *i* between two phases, octanol (*O*), and water (*W*) at infinite dilution, is measured with partition coefficient as follows^31^:

11 $$K_{i}^{O,W} = \frac{{x_{i}^{O} }}{{x_{i}^{W} }} = \frac{{\gamma_{i}^{W} }}{{\gamma_{i}^{O} }}$$

Therefore, the octanol/water partition coefficient for component *i* ($K_{OW,i}$) could be defined as follows^31^:

12 $$\log K_{OW,i} = \log \left( {\frac{{C_{o,W} \gamma_{i}^{W,\infty } }}{{C_{o,O} \gamma_{i}^{O,\infty } }}} \right)$$

where $C_{o,O}$ and $C_{o,W}$ are the total concentrations in octanol-rich and water-rich phases, respectively. The $\gamma_{i}^{O,\infty }$ and $\gamma_{i}^{W,\infty }$ are the activity coefficients of component *i* in octanol-rich and water-rich phases at dilute concertation. The default value for $\frac{{C_{o,W} }}{{C_{o,O} }}$ is 0.151. The octanol-rich phase is composed of 27.5 mol % water and 72.5 mol % octanol. The water-rich phase is free of octanol. Pharmaceutical compounds with high octanol–water partition coefficients are found mainly in hydrophobic areas, such as lipid bilayers of cells, and are called hydrophobic drugs. In contrast, hydrophilic drugs with low octanol/water partition coefficients are distributed in aqueous regions, such as blood serum.

### Predictive PC-SAFT EOS

To define predictive PC-SAFT EOS through COSMO calculations, five parameters of PC-SAFT EOS must correlate to COSMO results. In order to attain this objective, three steps were followed as below:

*Step 1* Constructing a relation between the segment number (*m*) and the segment diameter ($\sigma$) with the surface area of the cavity (*A*) and the total volume of the cavity (*V*) was obtained via the COSMO file. The following two equations could be written based on definitions for the surface area of the cavity^32^ and the total volume of the cavity^27^:

13 $$V = \frac{\pi }{6}m\sigma^{3}$$

14 $$A = \pi m\sigma^{2}$$

By incorporating two equations, the segment diameter ($\sigma$)for PC-SAFT EOS is calculated based on the COSMO result (the surface area of the cavity (*A*) and total volume of the cavity (*V*)) as follows:

15 $$\sigma = \frac{6 \, V}{A}$$

Knowing the segment diameter, the segment number (*m*) for PC-SAFT EOS is obtained as follows:

16 $$m = \frac{A}{{\pi \sigma^{2} }}$$

**Figure 2:**
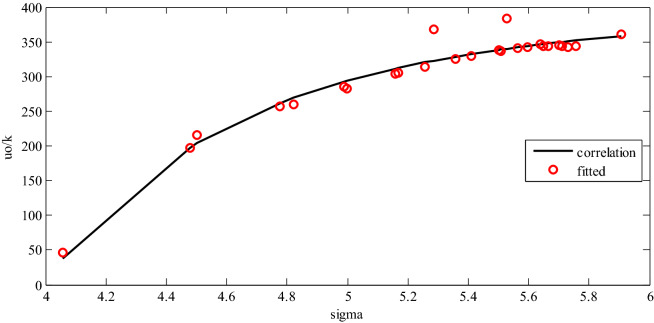
The fitted dispersion energies (symbol) versus correlated values from Eq. 17 (line).

*Step 2* In this step, a new methodology was applied to obtain the third PC-SAFT EOS parameter (dispersion energy ($u_{o}$)). Primarily, COSMO results consist of the surface area of the cavity and the total volume of the cavity comprises 26 non-associating compounds, which were mainly alkanes, methane to eicosane, alkenes, and cycloalkanes. The segment number (*m*) and the segment diameter ($\sigma$) for the considered compounds were calculated with Eqs. 15 and 16. By specifying two PC-SAFT EOS parameters, *m* and $\sigma$, dispersion energies were fitted for all the 26 surveyed non-associating compounds employing vapor pressure data. The vapor pressure data for all of the examined compounds were obtained from the correlations in a study by Reid et al.^30^. Van Nhu et al.^23^ suggested an inverse relation between dispersion energy and segment diameter ($u_{o} \propto \frac{1}{{\sigma^{6} }}$). To investigate this relation, the tuned dispersion energies were plotted versus segment diameters. According to Fig. 2, a linear relation existed between dispersion energy and $\sigma^{ - 6}$, which could be written as follows:

17 $$\frac{{u_{o} }}{k}\left[ K \right] = \frac{{ - 1.5979 \times 10^{6} }}{{\sigma \left( {{\dot{\text{A}}}} \right)^{6} }} + 396.143$$

Subsequently, the PC-SAFT EOS parameters for non-associating compounds *m*, $\sigma$, and $u_{o}$ were determined based on Eqs. 15–17.

*Step 3* Two additional PC-SAFT EOS parameters for associating compounds were found to be association energy ($\varepsilon^{AB}$) and association volume ($\kappa^{AB}$). They were identified in this step. The association volume values ranged between 0.01 and 0.03, and its variations had a negligible influence on the results. Therefore, the association volume proposed to withdraw from adjustable variables by assuming a constant value^33^. In this work, the constant 0.02 was set for its value.

For all the examined associating compounds, the association scheme 2B was assumed in order to make computations faster and cheaper. To determine a correlation for association energy, 13 associating compounds consisting of alkanols, methanol to octanol, and some amines were considered, whose COSMO calculations were done in this study. The segment numbers, segment diameters, and dispersion energies for 13 associating compounds were obtained via Eqs. 15–17. As described above, association volumes for 13 examined compounds were assumed to be 0.02. Afterwards, by knowing four PC-SAFT EOS parameters, association energy was tuned for each compound by comparing them to experimental vapor pressures obtained by Reid et al.^30^. In a procedure similar to dispersion energy, the fitted association energies were correlated with the segment diameter as follows:

18 $$\frac{{\varepsilon^{AB} }}{k}\left[ K \right] = \frac{{8.1418 \times 10^{6} }}{{\sigma \left( {{\dot{\text{A}}}} \right)^{6} }} + 1923.2$$

**Figure 3:**
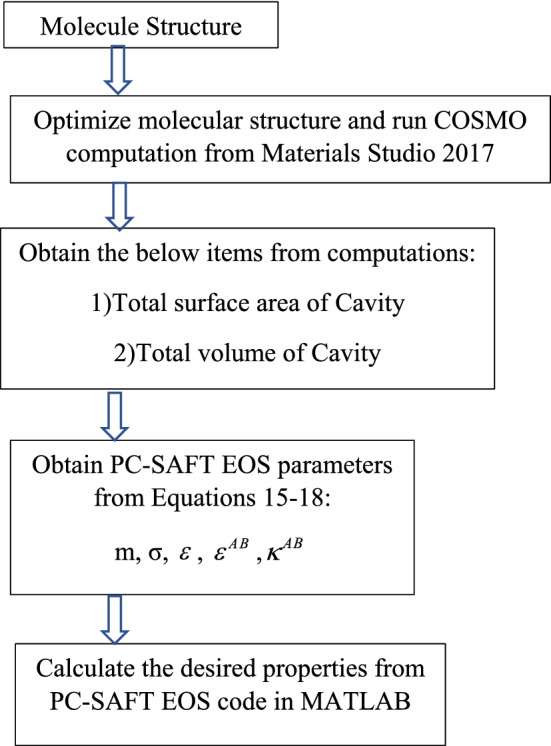
A schematic flow diagram of the outlined method.

Following this, the predictive PC-SAFT EOS was performed by incorporating Eqs. 15–18 into the original PC-SAFT EOS. In the predictive PC-SAFT, the adjustable parameters of the model were obtained via Eqs. 15–18 without further requirements to tune them with the experimental data. Figure 3 displays a schematic view of implemented methodology in this study.

### COSMO-RS

The conductor-like screening model for real solvents (COSMO-RS) was firstly introduced by Klamt^5^. In COSMO-RS, molecules are assumed as a collection of surface segments. Interaction energy between segments are computed based on COSMO calculations. In COSMO-RS, an expression for the molecule chemical potential is derived based on interaction energies between the segments in the condensed phase.

In COSMO-based models, the activity coefficient of component *i* in solvent *S* ($\gamma_{i,S}$) is reflective of two contributions: combinatorial part ($\gamma_{i,s}^{C}$) and residual part($\gamma_{i,s}^{R}$), as follows^7,8^:

19 $$\ln \gamma_{i,S} = \ln \gamma_{i,s}^{C} + \ln \gamma_{i,s}^{R}$$

The differences between the size and shape of solute and solvent are accounted in the combinatorial part and calculated with the Staverman-Guggenheim term as follows^34^:

20 $$\ln \gamma_{i,s}^{C} = \ln \frac{{\phi_{i} }}{{x_{i} }} + \frac{z}{2}q_{i} \ln \frac{{\theta_{i} }}{{\phi_{i} }} + l_{i} - \frac{{\phi_{i} }}{{x_{i} }}\sum\limits_{j} {x_{j} l_{j} }$$

where $\theta_{i}$, $\phi_{i}$ and $l_{i}$ are defined as follows:

21 $$\theta_{i} = \frac{{x_{i} q_{i} }}{{\sum\limits_{i} {x_{i} q_{i} } }};\phi_{i} = \frac{{x_{i} r_{i} }}{{\sum\limits_{i} {x_{i} r_{i} } }};l_{i} = \frac{z}{2}\left( {r_{i} - q_{i} } \right) - (r_{i} - {1})$$

In the above-mentioned expressions, $q_{i}$ and $r_{i}$ are respectively the normalized area and volume of molecules, and correlate with the total volume of cavity ($V_{i}$) and total surface area of cavity ($A_{i}$) as follows:

22 $$r_{i} = \frac{{V_{i} }}{{r_{o} }};q_{i} = \frac{{A_{i} }}{{q_{o} }}$$

where $r_{o}$ and $q_{o}$ are respectively defined based on the volume and area of an ethylene molecule as the normalization factor. The residual part of the COSMO-RS was defined as follows^6,7^:

23 $$\ln \gamma_{i,s}^{R} = \frac{{\mu_{i,S}^{{}} - \mu_{i,i} }}{RT}$$

In the expression above, *T* and *R* are system temperature and the universal gas constant, respectively. Herein, $\mu_{i,S}^{{}}$ and $\mu_{i,i}$ represent chemical potential of solute *i* in liquid solvent *S* and chemical potential of pure solute *i*, respectively. $\mu_{i,S}^{{}}$ could be obtained from the following equation:

24 $$\mu_{i,S}^{{}} \left( \sigma \right) = - RT\ln \left[ {d\sigma^{\prime} \, P\left( {\sigma^{\prime}} \right){\text{ exp}}\left( {\frac{{\mu_{i,S}^{{}} \left( {\sigma^{\prime}} \right) - a_{eff} \Delta W\left( {\sigma ,\sigma^{\prime}} \right)}}{RT}} \right)} \right]$$

The potential energy in the previous equation appears in both sides and must be determined by iteration. In Eq. 24, the exchange energy $\Delta W\left( {\sigma ,\sigma^{\prime}} \right)$ is defined as in:

25 $$\Delta W\left( {\sigma ,\sigma^{\prime}} \right) = \left( {\frac{{\alpha^{\prime}}}{2}} \right)\left( {\sigma + \sigma^{\prime}} \right)^{2} + c_{hb} \max \left[ {0,\sigma_{acc} - \sigma_{hb} } \right]\min \left[ {0,\sigma_{don} + \sigma_{hb} } \right]$$

The first term in the above-mentioned expression $\left( {\alpha^{\prime}} \right)$ shows the misfit energy. The $c_{hb}$ and $\sigma_{hb}$ are the energy-type constant and cutoff value for hydrogen bonding interaction^32^, respectively. The $\sigma_{acc}$ and $\sigma_{don}$ are respectively the maximum and minimum values of $\sigma$ and $\sigma^{\prime}$. In Eq. 24, the sigma distribution $\left( {P\left( {\sigma^{\prime}} \right)} \right)$ is computed based on the method outlined by Mahmoudabadi and Pazuki^16^. The default values of the mentioned constants were reported in Klamt et al.^35^.

### COSMO-SAC

The conductor-like screening models-segment activity coefficient (COSMO-SAC) was proposed by Lin and Sandler^8^. In COSMO-SAC, the activity coefficient is calculated through the solvation free energy of molecules in a solution in a two-step process: (i) the dissolution of the solute into a perfect conductor and (ii) the restoration of the conductor to the actual solvent. The residual part of the COSMO-SAC was defined as follows^8,36^:

26 $$\ln \gamma_{i,s}^{R} = \frac{{A_{i} }}{{a_{eff} }}\sum\limits_{\sigma } {P_{i} (\sigma )\left[ {\ln (\Gamma_{S} (\sigma )) - \ln (\Gamma_{i} (\sigma ))} \right]}$$

where $\Gamma (\sigma )$ is the segment activity coefficient and is calculated via the iteration according to the following equation:

27 $$\ln (\Gamma_{i} (\sigma )) = - \ln \left\{ {\sum\limits_{{\sigma_{n} }} {P_{i} (\sigma^{\prime})\Gamma_{i} (\sigma^{\prime})\exp \left[ { - \frac{{\Delta W(\sigma ,\sigma^{\prime})}}{RT}} \right]} } \right\}$$

## Results and discussions

The Predictive PC-SAFT EOS parameters for all the 35 considered pharmaceutical compounds and 15 solvents were obtained employing Eqs. 15–18. Table 2 represents a list of the calculated parameters on top of COSMO results for all the 80 examined compounds. Comparing the obtained predictive PC-SAFT EOS parameters to the original PC-SAFT EOS by Gross and Sadowski^27^, we observed that the predictive PC-SAFT EOS has a lower segment number and larger segment diameter. The dispersion energies obtained from Eq. 17 had larger values with respect to dispersion energies reported by Gross and Sadowski^27^. With the increase in the molecule volume and molar mass, both the segment number and segment diameter were enhanced, which obeys the same trend as by Gross and Sadowski^27^. The increment of molecule size for each hydrogen bonding site reduced association energies while Gross and Sadowski^28^ presented various trends for association energy.

**Table 2:** COSMO file results and predictive PC-SAFT EOS parameters.

	$V\left[ {bohr^{3} } \right]$	$A\left[ {bohr^{2} } \right]$	$m$	$\sigma (\dot{A})$	$\frac{{u_{o} }}{k}[K]$	$\frac{{\varepsilon^{AB} }}{k}[K]$
Methane	257.18	201.23	1.09	4.06	38.25	
Ethane	393.14	277.22	1.22	4.5	204.4	
Propane	523.59	344.65	1.32	4.82	269.27	
Butane	659.11	418.63	1.49	5	293.75	
Pentane	789.52	485.01	1.62	5.17	312.32	
Hexane	928.64	560.78	1.81	5.26	320.52	
Heptane	1055.55	625.43	1.94	5.36	328.66	
Octane	1194.86	701.06	2.13	5.41	332.52	
Nonane	1325.04	764.61	2.25	5.5	338.56	
Decane	1463.97	843.64	2.48	5.51	339.03	
Undecane	1587.51	905.74	2.61	5.57	342.35	
Dodecane	1721.47	976.56	2.78	5.6	344.17	
Tridecane	1847.83	1040.29	2.92	5.64	346.49	
Tetradecane	1987.15	1117.02	3.12	5.65	346.94	
Pentadecane	2117.19	1186.78	3.3	5.66	347.76	
Hexadecane	2256.59	1256.76	3.45	5.7	349.6	
Heptadecane	2391.79	1329.63	3.63	5.71	350.11	
Octadecane	2521.29	1397.32	3.79	5.73	350.95	
Nonadecane	2615.94	1406.47	3.59	5.91	358.47	
Eicosane	2787.1	1537.35	4.14	5.76	352.21	
Cyclopentane	696.5	418.38	1.33	5.29	322.87	
Cyclohexane	825.5	474.19	1.38	5.53	340.11	
Ethylene	361.18	256.13	1.14	4.48	197.82	
Propylene	496.9	330.23	1.29	4.78	261.78	
1-Butene	628.25	399.74	1.43	4.99	292.66	
1-Pentane	762.09	469.08	1.57	5.16	311.33	
Methanol	325.95	242.17	1.18	4.27	133.81	3259.86
Ethanol	451.88	309.34	1.28	4.64	235.65	2740.95
1-Propanol	589.87	376.5	1.36	4.97	290.68	2460.56
Propanol	589.87	376.5	1.36	4.97	290.68	2460.56
Butanol	728.92	456.11	1.58	5.07	302.52	2400.22
Pentanol	861.53	525.69	1.73	5.2	315.64	2333.38
Hexanol	990.95	593.37	1.88	5.3	324.25	2289.49
Heptanol	1125.29	664.31	2.05	5.38	330.13	2259.57
Octanol	1259.94	723.04	2.11	5.53	340.44	2207.04
Nonanol	1393.97	805.34	2.38	5.5	338.15	2218.69
Acetic Acid	486.15	327.88	1.32	4.71	249.35	2671.17
Methylamine	352.12	256.53	1.2	4.36	162.95	3111.37
Ethylamine	487.31	329.28	1.33	4.7	247.71	2679.54
Aniline	819.2	478.9	1.45	5.43	333.89	2240.39
Acetone	559.41	364.53	1.37	4.87	276.74	2531.58
Acetonitrile	430.47	296.24	1.24	4.61	230.47	2767.36
Ethyl Acetate	763.62	462.23	1.5	5.25	319.43	2314.09
Methyl Acetate	629.03	402.25	1.45	4.97	289.49	2466.62
2-Phenylacetamide.Mat'	1101.04	617.34	1.72	5.66	347.69	2170.1
4-Methylphthalic Anhydride	1159.78	640	1.72	5.75	352.1	2147.61
Aceclofenac	2387.18	1196.68	2.66	6.33	371.39	2049.31
Acetaminophen	1168.97	651.08	1.79	5.7	349.58	2160.44
Acetylsalicylic Acid	1309.21	698.84	1.76	5.95	360.07	2107.02
Atenolol	2130.34	1130.51	2.81	5.98	361.31	2100.68
Atropine	2191.67	1043.83	2.09	6.67	377.94	2015.95
Benzamide	972.21	549.81	1.55	5.61	345.12	2183.18
Camphor	1249.82	642.07	1.5	6.18	367.47	2069.29
Capecitabine	2526.62	1316.78	3.16	6.09	364.89	2082.43
Cefixime	2967.42	1477.68	3.24	6.38	372.36	2044.37
Celecoxib	2510.77	1274.61	2.9	6.25	369.45	2059.23
Cephalexin	2460.67	1208.32	2.58	6.47	374.28	2034.62
Cimetidine	1972.62	1059.47	2.7	5.91	358.71	2113.95
Dapsone	1772.55	913.98	2.15	6.16	366.83	2072.56
Deferiprone	1066.02	590.35	1.6	5.73	351.16	2152.42
Flurbiprofen	1838.12	952.08	2.26	6.13	366.03	2076.65
Hydroquinone	851.14	495.86	1.49	5.45	335.16	2233.91
Isoniazid	1049.15	594.69	1.69	5.6	344.41	2186.79
Lamotrigine	1652.5	860.9	2.07	6.09	364.96	2082.08
Meclofenamic Acid	1993.66	1001.39	2.23	6.32	371.1	2050.82
Pentoxifylline	2066.17	1082.17	2.62	6.06	363.95	2087.25
Pindolol	1987.66	1053.1	2.61	5.99	361.65	2098.98
P-Nitrobenzamide	1168.2	648.82	1.77	5.72	350.36	2156.47
Probenecid	2260.95	1135.48	2.53	6.32	371.12	2050.7
Sulfamethazine	1976.05	1009.33	2.33	6.22	368.45	2064.32
Vinpocetine	2641.37	1261.17	2.54	6.65	377.66	2017.36
Benzocaine	1298.88	717.38	1.93	5.75	351.87	2148.76
Borneol	1290.13	651.63	1.47	6.29	370.25	2055.15
Carvedilol	3077.3	1549.79	3.48	6.3	370.7	2052.86
Ibuprofen	1735.47	908.34	2.2	6.07	364.08	2086.57
Isoborneol	1288.31	652.16	1.48	6.27	369.9	2056.92
Salicylic Acid	1010.93	567.34	1.58	5.66	347.42	2171.46
Sulfacetamide	1470.16	774.99	1.9	6.02	362.68	2093.72
Trifloxystrobin	2917.8	1472.78	3.32	6.29	370.35	2054.63

**Figure 4:**
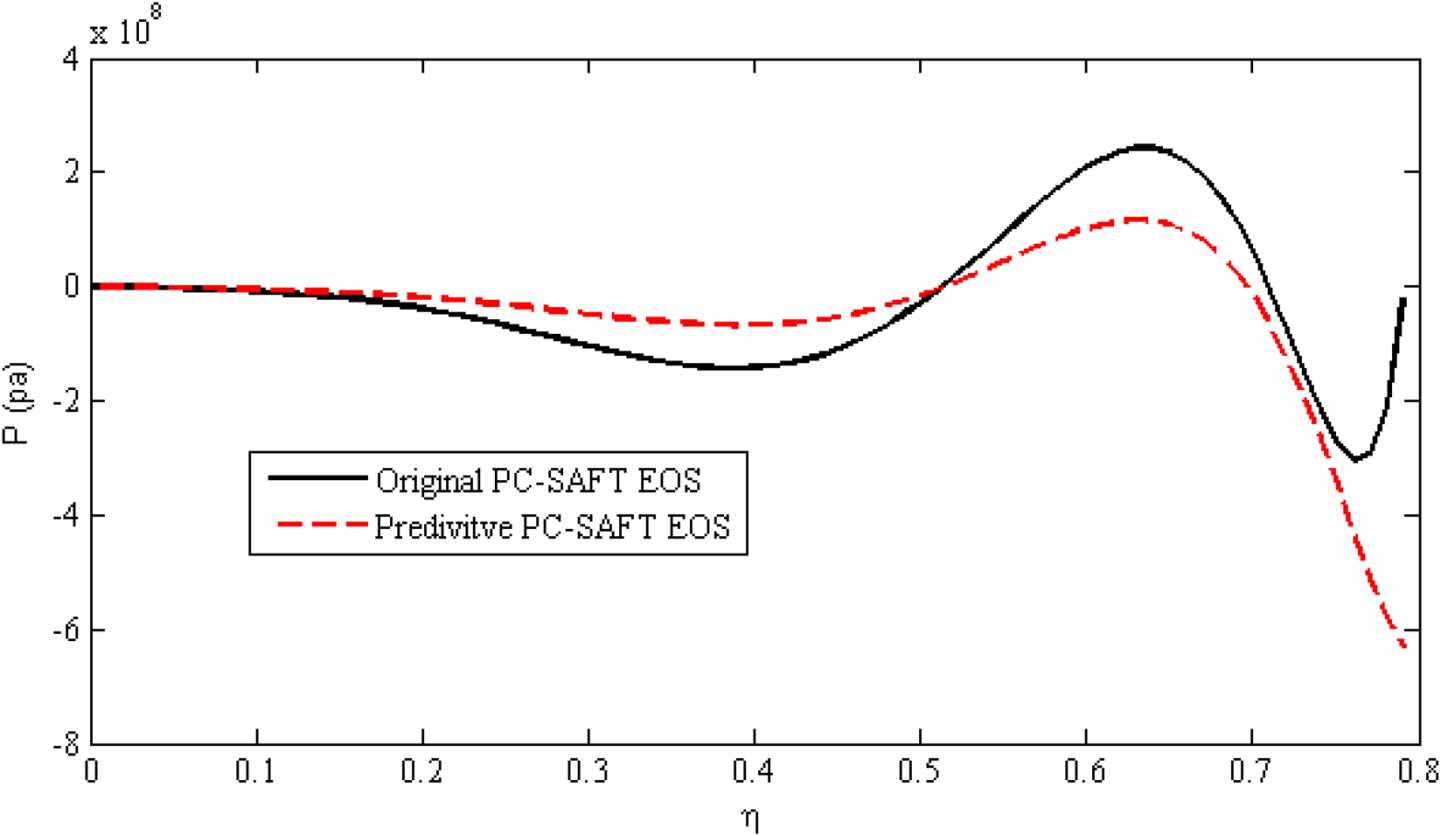
$P - \eta$ plane of n-decane at T = 135 K from PC-SAFT EOS (solid line) and predictive PC-SAFT EOS (dashed line).

Polishuk^37^ demonstrated the practically unrealistic and even nonphysical predictions of different version of SAFT EOS, such as PC-SAFT EOS at certain conditions. Privat et al.^38^ exhibited that the PC-SAFT EOS may have up to five different volume roots, some of which are not realistic. To test the above-mentioned statement for predictive PC-SAFT EOS, we followed the same approach as discussed in Privat et al.^38^. The $P - \eta$ plane of n-decane at T = 135 K for PC-SAFT EOS and predictive PC-SAFT EOS were plotted at Fig. 4. According to this figure, for a pressure close to zero, the predictive PC-SAFT had three volumetric roots compared to five roots of PC-SAFT EOS. This implied that reparameterization in the predictive PC-SAFT have reduced unrealistic roots of the PC-SAFT EOS.

**Figure 5:**
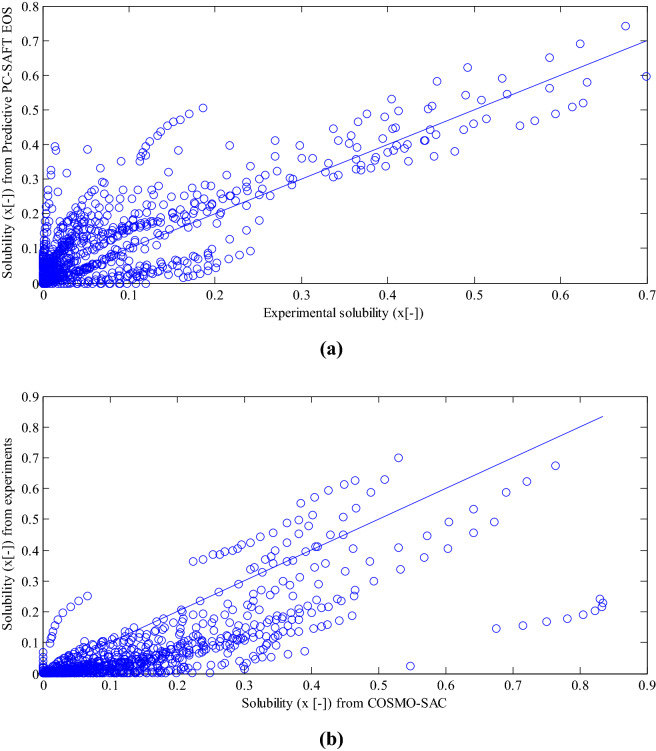
.(a) Parity plot of experimental solubility data (mole fraction) in pure solvents compared to calculated solubility data (mole fraction) from predictive PC-SAFT EOS results (b) Parity plot of experimental solubility data (mole fraction) in pure solvents compared to calculated solubility data (mole fraction) from COSMO-SAC model^16^ .

Afterwards, to examine the reliability of the obtained PC-SAFT parameters, experimental solubility data of all the 35 surveyed pharmaceutical compounds in 15 pure solvents were simulated with PC-SAFT EOS. For solubility in the binary system, 918 data for 110 systems over the temperature range of 262–360 K were explored. The mole fractions of the considered solubility data varied from $1 \times 10^{ - 7}$ to 0.7. Figure 5 depicts the parity plot of experimental solubility data in comparison with the solubilities from Predictive PC-SAFT EOS (a) and COSMO-SAC model (b). Table 3 reports RMSEs based on logarithmic scale for predictive PC-SAFT EOS, COSMO-SAC, and COSMO-RS models. The predictive PC-SAFT EOS was of object in the current study. The COSMO-SAC results were derived from Mahmoudabadi and Pazuki ^16^. The COSMO-RS results were obtained in this study utilizing COSMOtherm software. Based on Fig. 5 and Table 3, the predictive PC-SAFT EOS showed a good representation of experimental data. In all of the examined systems, binary interaction parameters were set zero. The root-mean-square-error (RMSE) based on logarithmic scale for the predictive PC-SAFT EOS was 1.435. The calculated RMSE for the predictive PC-SAFT EOS was less than the reported RMSE (4.389) for COSMO-SAC by Mahmoudabadi and Pazuki^16^. The RMSEs for COSMO-RS model was 1.412. According to Table 3, the predictive PC-SAFT EOS and COSMO-RS model represented a same accuracy of experimental data. For example, the predictive PC-SAFT EOS for acetyl salicylic acid, celecoxib, and hydroquinone has the better performance compared to COSMO-SAC and COSMO-RS. The molecules containing H, C, and O atoms provided better estimations of solubility data without further complexity in atoms bonds. In any quantum chemistry software package, various density function theories are afforded for computing the COSMO file, which have different and almost unpredictable accuracy for each specific type of molecule. In order to asset better approximation of the desired properties for a specified molecule, the accuracy of each density function theories must be analyzed. Furthermore, default atomic radii inputs to software based on COSMO calculations have certain uncertainty and also the flexibility to justify their values in an approach similar to that suggested by Van Nhu et al.^23^

.

**Table 3:** RMSEs for predictive PC-SAFT EOS compared to RMSEs for COSMO-SAC and COSMO-RS for investigated systems.

Drug	Solvent	RMSE for COSMO-SAC	RMSE for Predictive PC-SAFT	RMES for COSMO-RS
Acetaminophen	Ethanol	0.4207	0.2333	0.0792
Acetaminophen	2-propanol	0.945	0.2921	0.2577
Acetaminophen	Octanol	0.86	0.7975	0.2900
Acetyl salicylic acid	Ethanol	0.1321	0.1543	0.2073
Acetyl salicylic acid	2-propanol	0.6646	0.2626	0.5881
Acetyl salicylic acid	Octanol	0.2871	0.1597	0.3529
Atenolol	Hexane	3.0371	7.869	1.6205
Atenolol	Octanol	0.2576	0.8698	0.3247
Atropine	Ethanol	0.0808	0.5162	0.4052
Atropine	Octanol	0.3231	0.574	0.2488
Celecoxib	Water	5.5585	1	0.9229
Celecoxib	2-propanol	3.0148	0.3367	0.9661
Cimetidine	Methanol	1.3713	0.6608	1.2136
Cimetidine	Ethanol	1.8764	1.6795	1.5665
Dapsone	Acetone	37.9528	0.9605	0.5671
Dapsone	Methyl acetate	0.3239	0.4535	1.4856
Dapsone	Methanol	0.7903	1.6803	1.2825
Dapsone	Ethanol	2.0572	2.3074	1.3358
Hydroquinone	Ethyl acetate	229.1155	1.3882	1.446
Hydroquinone	Acetic acid	1.1542	0.1534	0.8517
Hydroquinone	Methanol	3.8924	1.7398	0.7672
Hydroquinone	Ethanol	3.476	1.9527	0.3403
Isoniazid	Ethanol	1.6081	1.9557	1.4499
Isoniazid	Methanol	1.4152	0.9793	1.3168
Isoniazid	Ethyl acetate	3.6146	2.8687	3.5192
Isoniazid	Acetone	1.6601	1.1512	1.5884
Lamotrigine	Water	2.1866	1	0.7806
Lamotrigine	Methanol	3.297	1.8426	2.7684
Lamotrigine	Ethyl acetate	3.6465	0.5192	2.9723
Lamotrigine	Acetonitrile	4.0032	1	7.241
Meclofenamic acid	Water	0.8871	0.3776	0.9219
Meclofenamic acid	Ethanol	1.3238	0.6631	1.3181
Meclofenamic acid	Octanol	1.1912	1	3.2589
Pentoxifylline	Water	1.8649	1.837	1.2719
Pentoxifylline	Ethanol	1.5273	1.2159	3.7059
Pentoxifylline	Octanol	2.3665	1.5075	0.9401
pNitrobenzamide	Ethanol	0.6927	1	1.802
pNitrobenzamide	Water	3.4488	0.7366	2.538
pNitrobenzamide	Ethyl acetate	2.3136	2.1542	1.7365
pNitrobenzamide	Acetonitrile	1.2261	0.4587	0.0437
Probenecid	Methanol	0.7749	1.1843	0.1434
Probenecid	Acetone	1.9154	0.7897	4.3467
Probenecid	Ethyl acetate	1.3842	5.2666	1
Pindolol	Hexane	0.7479	0.7928	1.701
Pindolol	Octanol	1.4195	1.3764	0.1201
Salicylic acid	Acetone	2.0516	1.2808	0.4577
Salicylic acid	Ethyl acetate	0.1844	1.1409	0.142
Salicylic acid	Methanol	5.062	1	3.9941
Salicylic acid	Water	0.3336	0.1487	0.43
Salicylic acid	Acetic acid	1.3766	0.8741	0.2092
Sulfacetamide	Ethanol	1.1653	1	2.8807
Sulfacetamide	Water	2.7188	2.9539	1.8547
Trifloxystrobin	Propanol	2.4643	3.7978	1.8583
Trifloxystrobin	Heptane	2.124	3.9747	1.7845
Trifloxystrobin	Hexane	1.0965	0.6418	0.9391
Benzamide	Acetone	2.0342	1.4265	1.8752
Benzamide	Ethyl acetate	0.8995	1.78	0.9984
Benzamide	Acetonitrile	1.237	1.3013	1.2139
Benzamide	Methyl acetate	0.6833	0.5347	0.4988
Benzamide	Methanol	1.1192	0.9792	0.6971
Benzamide	Propanol	1.3039	1.1635	0.8812
Benzamide	2-propanol	3.2333	1	1.874
Benzamide	Water	0.7428	1.0028	0.7152
Borneol	Acetone	0.9895	1.1349	0.8962
Borneol	Ethanol	0.3303	0.1935	0.3383
Camphor	Acetone	0.1472	0.1421	0.2272
Camphor	Ethanol	0.1341	0.1629	0.3006
Isoborneol	Acetone	0.3473	0.0737	0.3648
Isoborneol	Ethanol	2.2225	0.189	1
Carvedilol	Acetone	3.8066	1.1928	1
Carvedilol	Ethyl acetate	3.3238	3.3796	1
Carvedilol	Methanol	1.2611	1	0.6244
Carvedilol	Ethanol	1.0957	0.4106	0.6802
Deferiprone	Water	1.4757	2.0661	1.4493
Deferiprone	Methanol	2.9636	3.0393	3.0312
Deferiprone	Acetonitrile	0.5252	0.3034	0.5592
Deferiprone	Ethyl acetate	0.2793	0.0834	0.3264
Ibuprofen	Ethanol	1.3427	4.3903	0.2677
Ibuprofen	Octanol	0.1364	0.8893	0.1326
Vinpocetine	Methanol	0.773	1	3.2242
Vinpocetine	Ethyl acetate	2.7753	1.9654	1.1719
Sulfamethazine	Water	1.8903	1.2592	1.1059
Sulfamethazine	Methanol	1.3965	1.2927	1.4223
Sulfamethazine	Acetonitrile	2.3546	2.1142	2.4297
2-phenylacetamide	Acetone	1.1625	0.9184	1.0492
2-phenylacetamide	Ethyl acetate	1.3422	1.4972	1.0658
2-phenylacetamide	Methanol	1.2167	1.3887	0.9893
2-phenylacetamide	Ethanol	1.4349	1.6067	1.2065
2-phenylacetamide	Propanol	0.3093	0.2228	0.2912
2-phenylacetamide	2-propanol	2.2784	2.8462	2.2277
4-methylphthalic anhydride	Acetone	0.0057	0.0342	0.0296
4-methylphthalic anhydride	Heptane	0.2161	0.2040	0.0541
4-methylphthalic anhydride	Methyl acetate	0.5383	2.1087	1.5019
4-methylphthalic anhydride	Acetonitrile	1.0519	2.0427	0.5273
Aceclofenac	Propanol	0.9884	0.1776	0.1474
Aceclofenac	Ethyl acetate	0.5593	0.1660	0.3481
Aceclofenac	Methanol	1.7541	0.3435	0.6107
Aceclofenac	Ethanol	1.4532	1.1943	0.8602
Aceclofenac	Propanol	2.3177	1.8202	1.7087
Capecitabine	Ethanol	4.7103	1	1.9346
Capecitabine	Propanol	8.9866	1	3.2988
Capecitabine	Water	7.0479	1.7073	2.1529
Cefixime Trihydrate	Water	6.8895	6.1175	6.6117
Cefixime Trihydrate	Methanol	16.6274	5.4366	7.5497
Cefixime Trihydrate	Acetonitrile	2.1109	1	5.5559
Cefixime Trihydrate	Ethyl acetate	4.9767	5.0494	0.5216
Cephalexin	Water	7.7391	8.0623	3.308
Cephalexin	Methanol	0.8088	0.3916	1
Cephalexin	Acetonitrile	0.4077	0.093	1
Flurbiprofen	Ethanol	4.38936	1.43514	1.4129
Flurbiprofen	Octanol	0.4207	0.2333	0.0792

**Figure 6:**
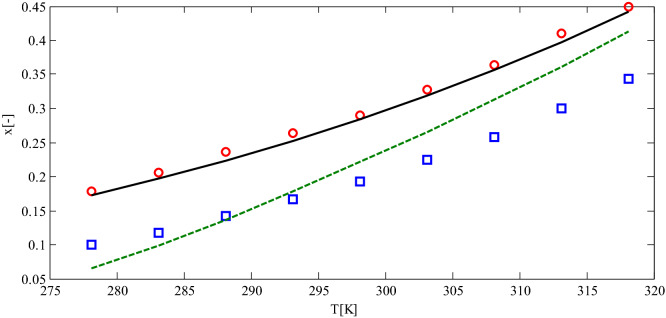
The predictive PC-SAFT EOS solubilities for 4-methylphthalic anhydride in methyl acetate (solid line) and acetonitrile (dashed line) in comparison to experimental data for solubility of 4-methylphthalic anhydride in methyl acetate (circle) and acetonitrile (square)^39^.

**Figure 7:**
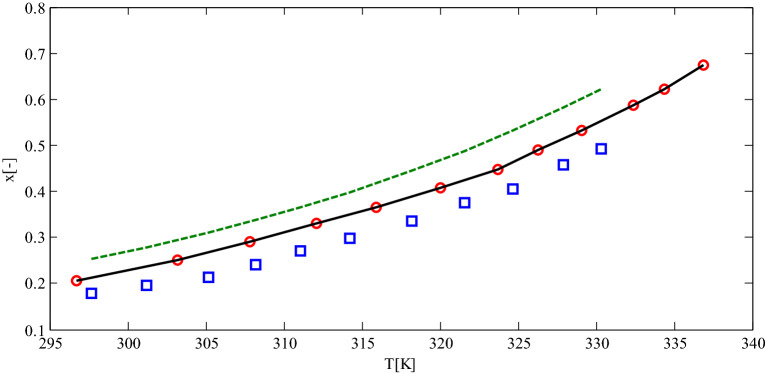
The predictive PC-SAFT EOS solubilities for ibuprofen in octanol (solid line) and ethanol (dashed line) in comparison to experimental data for solubility of ibuprofen in octanol (circle) and ethanol (square)^40^.

Figure 6 compares experimental solubility data of 4-methylphthalic anhydride in pure solvents of methyl acetate and acetonitrile with the results obtained by the predictive PC-SAFT EOS model. According to the figure, predictive PC-SAFT EOS represented a good match with experimental data. Figure 7 displays the predictive PC-SAFT EOS solubilities for ibuprofen in octanol and ethanol compared to experimental data for solubility. According to Fig. 7, a good agreement was observed between experimental data and model calculation for ibuprofen-octanol mixtures. At the same time, some deviations from experimental data were observed in ethanol-ibuprofen mixtures, which could be eliminated by introducing binary interaction parameters between ethanol and ibuprofen. According to Figs. 6 and 7, the predictive PC-SAFT EOS and experimental data have the same behaviors as temperature increments.

It could be concluded that the observed errors in predictive PC-SAFT were originated from different sources, including (i) various quantum chemistry theories manipulated in COSMO file generation, (ii) inaccuracy in values of atomic radii input for COSMO calculations, (iii) errors from neglecting binary interaction parameters in PC-SAFT EOS, and (iv) deficiencies in original PC-SAFT EOS theories.

**Figure 8:**
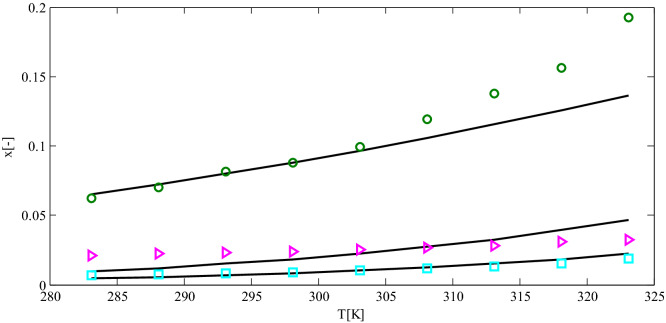
Calculated solubilities from the predictive PC-SAFT EOS (solid lines) for dapsone in pure acetone, methyl acetate, and methanol with the optimized binary interaction parameters -0.0455, 0.0156, and 0.0758, respectively, versus the experimental data of solubility for dapsone in pure acetone (circle), methyl acetate (triangular), and methanol (square)^41^.

For instance, the calculated dapsone solubilities in acetone, methyl acetate, and methanol from predictive PC-SAFT illustrated 50–60 AAD% compared to experimentally reported data. As described before, tuning a binary interaction parameter was suggested for correcting the reported error. Therefore, the binary interaction parameters between dapsone-acetone, dapsone-methyl acetate, and dapsone-methanol were fitted by justifying the experimental data and the following objective function:

28 $$ob = \sum {\left| {\frac{{x_{i,cal} - x_{i,\exp } }}{{x_{i,\exp } }}} \right|}$$

where $x_{i,cal}$ and $x_{i,\exp }$ were the calculated and experimental solubility of component *i*. The binary interaction parameters were regressed employing fminsearch algorithm in MATLAB programming software. The regressed binary interaction parameters for dapsone-acetone, dapsone-methyl acetate, and dapsone-methanol were − 0.0455, 0.0156, and 0.0758, respectively. The calculated solubilities of dapsone in acetone, methyl acetate, and methanol are plotted in Fig. 8. As could be observed, a good agreement was observed between model calculation and experimental data after incorporating binary interaction parameters. Thus, the absolute average percentage errors reduced from 50–60% to 16–18% by optimizing the binary interaction parameters. The binary interaction parameters in the proposed framework, the predictive PC-SAFT EOS, enhanced the predictivity character of the proposed model. In contrast, some other predictive models, such as COSMO-SAC and COSMO-RS, do not have this capability.

**Figure 9:**
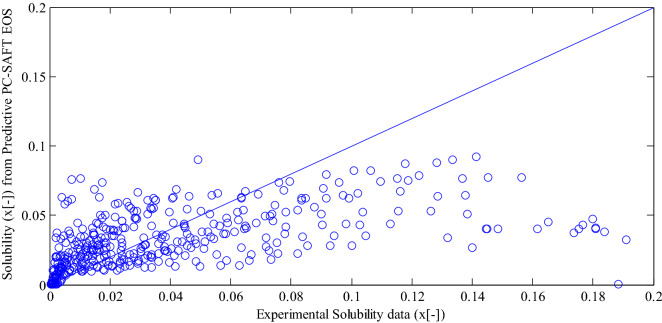
Parity plot of experimental solubility data (mole fraction) in binary solvents compared to calculated solubility data (mole fraction) from predictive PC-SAFT EOS results.

In addition to pure solvents, experimental solubility data in binary solvents was also tested. For solubility in the ternary system, 453 data for 10 systems over the temperature range of 277–325 K were explored. The mole fractions of the considered solubility data varied from $1 \times 10^{ - 4}$ to 0.2. Figure 9 demonstrates the parity plot of experimental solubility versus estimated solubilities from predictive PC-SAFT EOS. According to Fig. 9, a good accordance was observed between model calculations and experimental data. Certain deviations from experimental data were observed at the higher solubilities. In other words, the predictive PC-SAFT EOS underestimated the solubilities greater than 0.06. The RMSE based on logarithmic scale for the ternary system of pharmaceutical compounds in the cosolvent mixtures was 0.915. The computed RMSE is comparable to COSMO-SAC values (4.389) for the binary system of drug and solvent.

**Figure 10:**
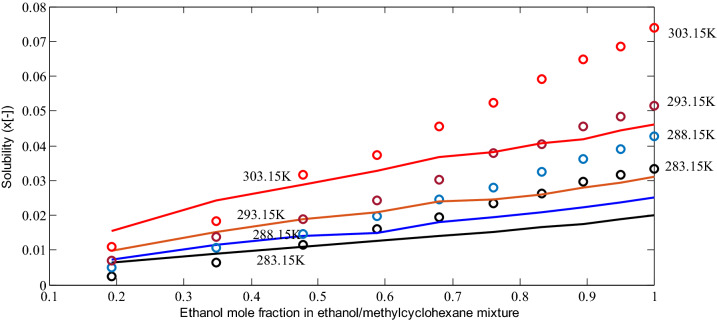
Calculated solubilities from predictive PC-SAFT EOS (line) versus experimental solubilities (circle) of acetylsalicylic acid in ethanol/methyl cyclohexane mixtures at temperatures 283.15, 288.15, 293.15. and 303.15 K^42^.

In Fig. 10, the calculated solubilities of acetyl salicylic acid in ethanol/methyl cyclohexane mixtures from predictive PC-SAFT EOS at four temperatures of 283.15, 288.15, 293.15, and 303.15 K are compared to experimental solubilities in the literature. According to the figure, at lower ethanol mole fractions, the predictive PC-SAFT reports a good representation of experimental data. Meanwhile, at higher mole fractions, model deviations from experimental data were observed. The observed deviations enhance with temperature increments, but model calculations and experiments follow a similar trend as function of temperature and mole fractions. The deviations at higher ethanol mole fraction could be corrected by fitting binary interaction parameters between ethanol and acetyl salicylic acid.

Octanol/water partition coefficients for all the surveyed pharmaceutical compounds were calculated by predictive PC-SAFT and compared with the reported experimental data in Table 4. Based on Table 4, the calculated octanol/water partition coefficients for some compounds, acetyl salicylic acid, celecoxib, and lamotrigine for instance, matched with experimental values. The predictive PC-SAFT estimated larger octanol/ water partition coefficients for some pharmaceutical compounds, such as aceclofenac, acetaminophen, and atenolol. This implied the higher hydrophobic nature of the considered pharmaceutical compounds employing the predictive PC-SAFT EOS. Camphor, flurbiprofen, meclofenamic acid, isoborneol, and salicylic acid indicated lower octanol/partition coefficient with respect to experimental values, which shows lower mole fraction of the examined pharmaceutical compounds in the octanol-rich phase than mole fraction in the water-rich phase. The standard RMSE for octanol/water partition coefficient of the investigated pharmaceutical compounds was 1.515.

**Table 4:** Octanol/water partition coefficients from predictive PC-SAFT in comparison to experimental data ^43^.

Substance	log K_OW,Predictve PC-SAFT_	log K_OW, exp_
2-phenylacetamide	1.05	0.45
Aceclofenac	3.61	2.17
Acetaminophen	1.19	0.46
Acetylsalicylic acid	1.42	1.19
Atenolol	3.06	0.16
Atropine	3.33	1.83
Benzamide	0.79	0.64
Camphor	1.31	2.38
Celecoxib	3.84	3.53
Cephalexin	3.80	0.65
Cimetidine	2.75	0.4
Dapsone	2.35	0.97
Flurbiprofen	2.48	4.16
Hydroquinone	0.63	0.59
Lamotrigine	2.1	2.57
Meclofenamic acid	2.82	5
Pentoxifylline	2.93	0.29
Pindolol	2.77	1.75
P-nitrobenzamide	1.18	0.82
Probenecid	3.35	3.21
Sulfamethazine	2.8	0.89
benzocaine	1.44	1.86
borneol	1.4	3.24
carvedilol	4.99	4.19
ibuprofen	2.27	3.97
isoborneol	1.39	3.24
salicylic acid	0.88	2.26
trifloxystrobin	4.66	4.5

## Conclusions

According to online literature, various attempts were made in order to attain a predictive-type PC-SAFT EOS. In predictive-type PC-SAFT EOS, the input parameters to the model are determined from another procedure instead of tuning with experimental data. Group contribution methods and correlation with molecular mass are a few examples. Among these approaches, developing a predictive model based on quantum chemistry calculation has become incredibly popular. The *ab* initio, Monte Carlo, and COSMO-based models are certain approaches to quantum chemistry calculations.

In the current study, quantum chemistry calculations based on COSMO were selected in order to make a predictive-type PC-SAFT EOS. The surface area of the cavity and total volume of the cavity were obtained with the COSMO file of 80 compounds consisting of pharmaceutical compounds, non-associating organic compounds, associating organic compounds, and popular solvents for pharmaceuticals. Two mentioned properties were incorporated into the adjustable input parameters for the PC-SAFT EOS by introducing some correlations based on COSMO results. Thus, a predictive PC-SAFT EOS was introduced utilizing the above-mentioned methods and correlations. To highlight the operability of the newly modified EOS, the solubility data of pharmaceutical compounds in pure/mixed solvents were simulated by the predictive PC-SAFT EOS and compared to COSMO-SAC and COSMO-RS models. According to the results of predictive PC-SAFT EOS, a good agreement was observed between model and experimental data in both binary and ternary systems. The predictive PC-SAFT EOS presented a better approximation of the experimental data when compared to COSMO-SAC model. The predictive PC-SAFT EOS and COSMO-RS model demonstrated the same accuracies. The observed deviations of predictive PC-SAFT from experimental data could be elaborated with the basic theory of applied density function, default values for atomic radii in COSMO calculations, the theory of PC-SAFT EOS, and elimination of binary interaction parameters. As in one case for dapsone, a good representation of experimental data with predictive PC-SAFT was concluded by fitting binary interaction parameters. It shed light to the fact that the volume and surface of molecules could be calculated employing any methods on top of COSMO, which is a crucial feature of the developed predictive PC-SAFT EOS.

## Supplementary Information

Supplementary Information
