# Supplementary material for: A predictive PC-SAFT EOS based on COSMO for pharmaceutical compounds
Source: Sci Rep. 2021 Mar 19;11:6405. doi: 10.1038/s41598-021-85942-8 (PMC7979706; doi:10.1038/s41598-021-85942-8)
Supplement: Supplementary file 1 — Supplementary Information [file 41598_2021_85942_MOESM1_ESM.pdf]

Supplementary materials

A Predictive PC-SAFT EOS Based on COSMO for Pharmaceutical Compounds

Samane Zarei Mahmoudabadi, Gholamreza Pazuki*

Department of Chemical Engineering, Amirkabir University of Technology (Tehran Polytechnic) Tehran, Iran

* Corresponding author. Tel.: + 98 21 64543159; fax: + 98 21 66405847; Email: ghpazuki@aut.ac.ir

Table S1. The melting point, enthalpy of fusion, and chemical structure for examined pharmaceutical compounds.

Name	Chemical Structure	T_m [K]	ΔH_m [J/mol]	Reference
2-Phenylacetamide	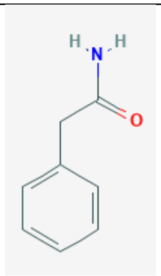	406.67	2.53E+04	[1]
4-Methylphthalic Anhydride	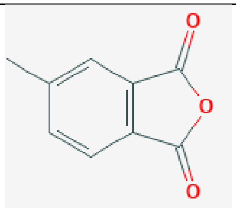	361.47	1.93E+04	[2]
Aceclofenac	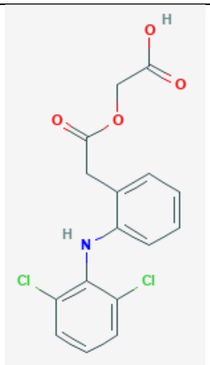	424.55	5.20E+04	[3]

Acetaminophen	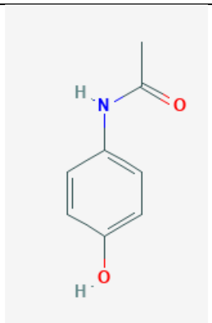	442.35	2.75E+04	[4]
Acetylsalicylic Acid	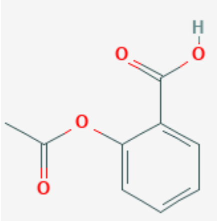	414.15	3.55E+04	[4]
Atenolol	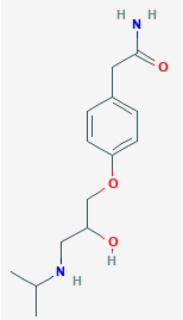	426.10	3.87E+04	[5]
Atropine	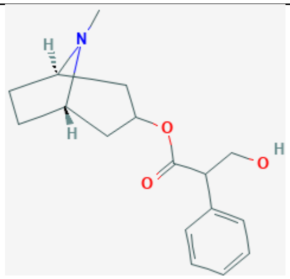	388.50	3.55E+04	[6]

Benzamide	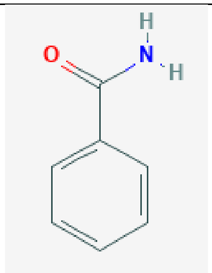	401.10	1.92E+04	[7]
Borneol	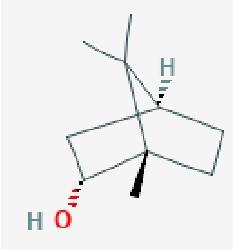	478.30	8.29E+03	[8]
Isoborneol	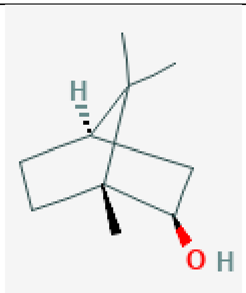	482.70	8.80E+03	[8]
Camphor	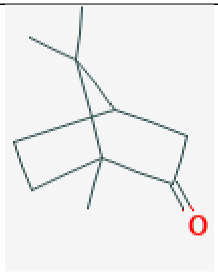	451.50	6.82E+03	[8]

Capecitabine	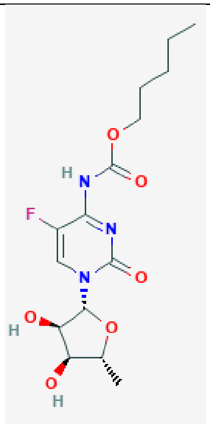	393.05	2.72E+04	[9]
Carvedilol	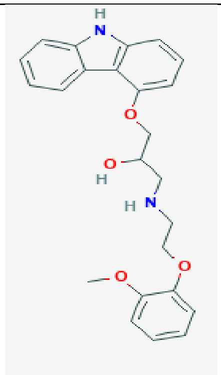	398.68	4.84E+04	[10]
Cefixime Trihydrate	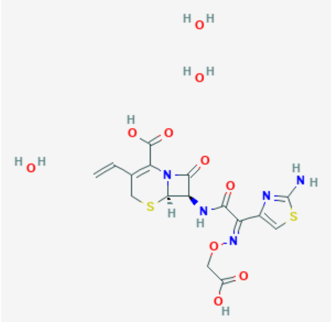	500.15	2.39E+04	[11]

Celecoxib	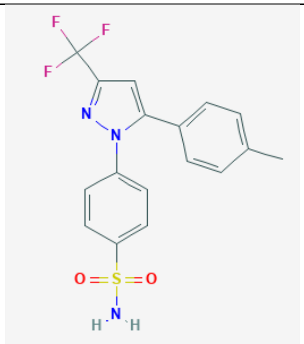	431.15	37908.178	[12, 13]
Cephalexin Monohydrate	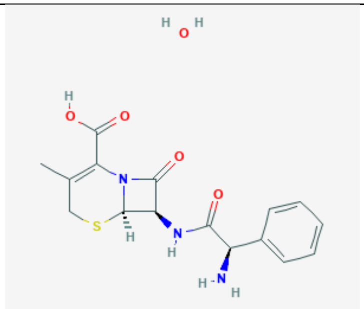	500.15	2.39E+04	[11]
Cimetidine	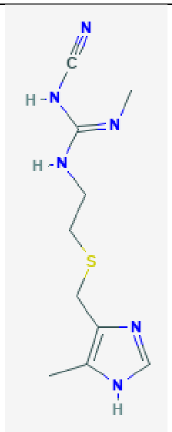	414.15	3.55E+04	[4]

Dapsone	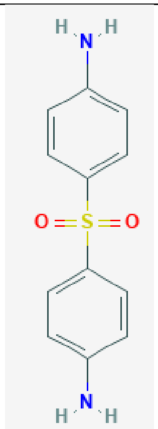	454.41	2.86E+04	[14]
Deferiprone	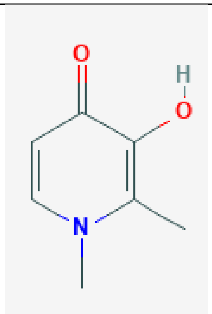	545.15	3.30E+04	[11]

flurbiprofen	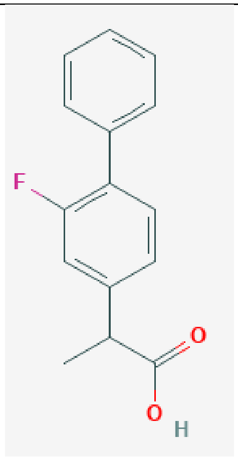	386.20	2.68E+04	[6]
Hydroquinone	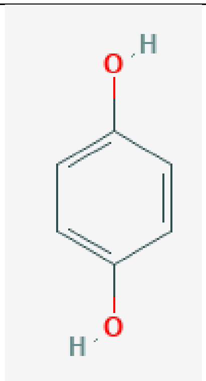	445.00	2.71E+04	[15]

Ibuprofen	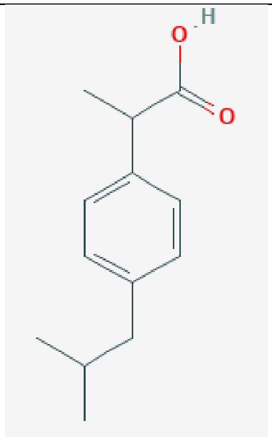	347.70	2.77E+04	[6]
Isoniazid	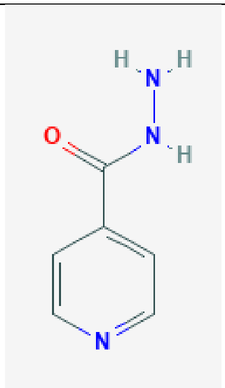	445.84	2.79E+04	[16]

Lamotrigine	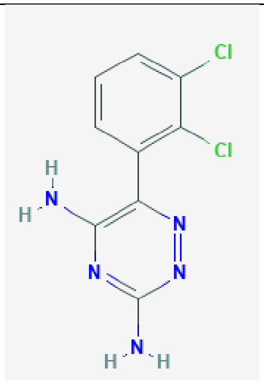	491.00	3.90E+04	[11]
Meclofenamic Acid	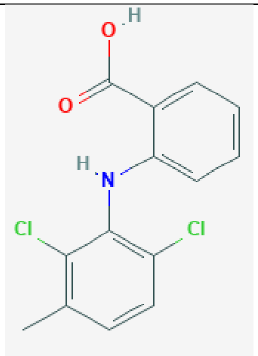	402.50	1.83E+04	[6]
Pentoxifylline	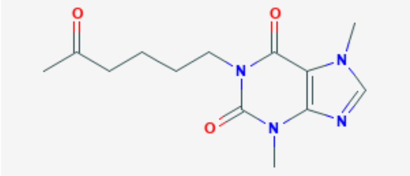	376.80	3.66E+04	[6]

P-nitrobenzamide	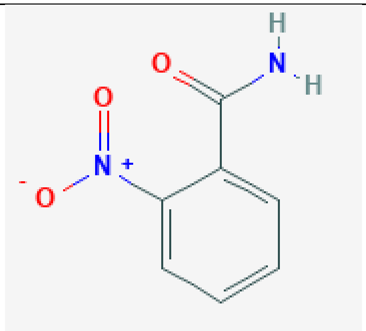	473.45	3.30E+04	[17]
Probenecid	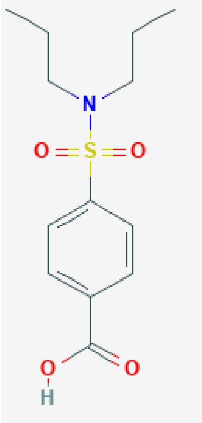	472.05	4.09E+04	[18]
Pindolol	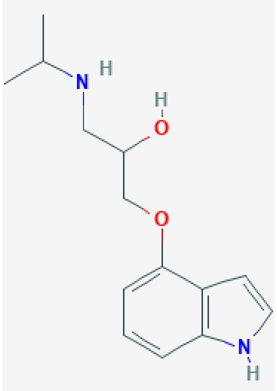	423.60	6.06E+04	[5]

Salicylic Acid	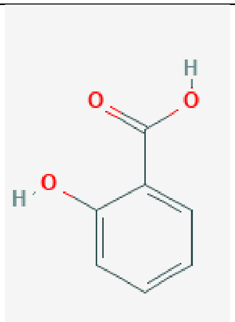	432.65	2.71E+04	[19]
Sulfacetamide	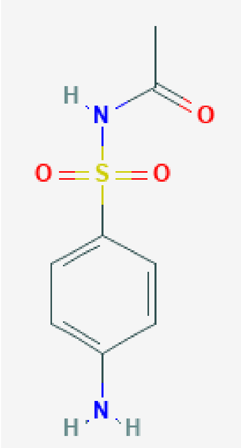	455.20	2.98E+04	[20]
Trifloxystrobin	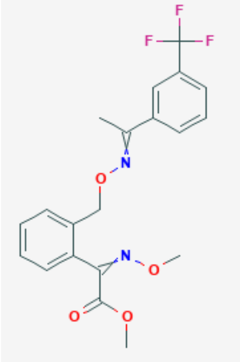	345.67	3.17E+04	[21]

Vinpocetine	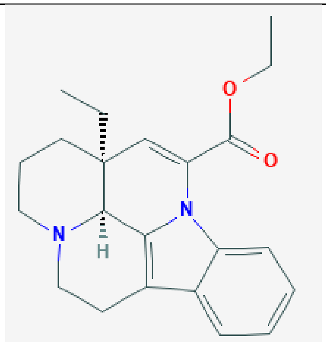	422.15	3.74E+04	[22]
Sulfamethazine	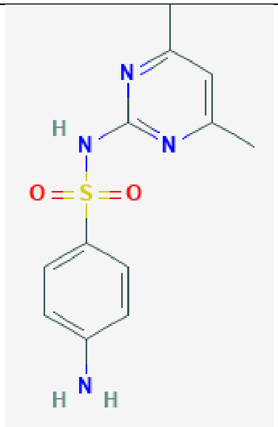 	469.20	3.77E+04	[23, 24]

Benzocaine	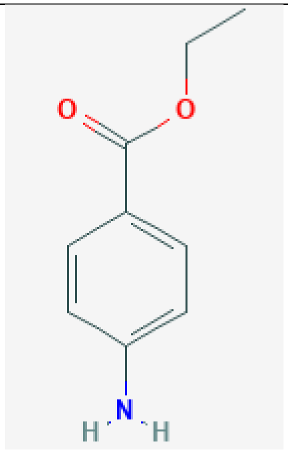	363.40	2.10E+04	[10]

Reference

1. Li, Y., K. Wu, and L. Liang, *Solubility behavior of 2-phenylacetamide in sixteen pure solvents and dissolution properties of solution.* Journal of Molecular Liquids, 2019. **291**. 10.1016/j.molliq.2019.111264.
2. Yu, Y., et al., *Experiment, correlation and molecular simulation for solubility of 4-methylphthalic anhydride in different organic solvents from T = (278.15 to 318.15) K.* Journal of Molecular Liquids, 2019. **275**: p. 768-777. 10.1016/j.molliq.2018.10.158.
3. Liu, J.Q., et al., *Experimental measurements and modeling of the solubility of aceclofenac in six pure solvents from (293.35 to 338.25) K.* Journal of Chemical and Engineering Data, 2014. **59**(5): p. 1588-1592. 10.1021/je500038u.
4. Matsuda, H., et al., *Determination and prediction of solubilities of active pharmaceutical ingredients in selected organic solvents.* Fluid Phase Equilibria, 2015. **406**: p. 116-123. 10.1016/j.fluid.2015.07.032.
5. Perlovich, G.L., T.V. Volkova, and A. Bauer-Brandl, *Thermodynamic study of sublimation, solubility, solvation, and distribution processes of atenolol and pindolol.* Molecular Pharmaceutics, 2007. **4**(6): p. 929-935. 10.1021/mp070039b.
6. Domañska, U., et al., *pKa and solubility of drugs in water, ethanol, and 1-octanol.* Journal of Physical Chemistry B, 2009. **113**(26): p. 8941-8947. 10.1021/jp900468w.
7. Ouyang, J., et al., *Solubility determination and modelling of benzamide in organic solvents at temperatures from 283.15 K and 323.15 K, and ternary phase diagrams of benzamide-benzoic acid cocrystals in ethanol at 298.15 K.* Journal of Molecular Liquids, 2019. **286**. 10.1016/j.molliq.2019.110885.
8. Chen, J., et al., *Determination and Correlation of Solubility of Borneol, Camphor, and Isoborneol in Different Solvents.* Journal of Chemical and Engineering Data, 2019. **64**(4): p. 1826-1833. 10.1021/acs.jced.9b00045.
9. Zhao, R., et al., *Solubility and dissolution characteristics of capecitabine in pure lower alcohols and water with methanol mixture solvents at atmospheric pressure and different temperatures.* Fluid Phase Equilibria, 2018. **460**: p. 23-35. 10.1016/j.fluid.2017.12.024.
10. Ha, E.S., et al., *Equilibrium solubility and solute-solvent interactions of carvedilol (Form I) in twelve mono solvents and its application for supercritical antisolvent precipitation.* Journal of Molecular Liquids, 2019. **294**. 10.1016/j.molliq.2019.111622.
11. Yousefi Seyf, J. and A. Haghtalab, *Measurement and thermodynamic modeling of the solubility of lamotrigine, deferiprone, cefixime trihydrate, and cephalexin monohydrate in different pure solvents from 283.1 to 323.1 K.* Journal of Chemical and Engineering Data, 2016. **61**(6): p. 2170-2178. 10.1021/acs.jced.6b00163.
12. Jouyban, A., S. Nozohouri, and F. Martinez, *Solubility of celecoxib in {2-propanol (1) + water (2)} mixtures at various temperatures: Experimental data and thermodynamic analysis.* Journal of Molecular Liquids, 2018. **254**: p. 1-7. 10.1016/j.molliq.2018.01.033.
13. Nozohouri, S., et al., *Solubility of celecoxib in N-methyl-2-pyrrolidone + 2-propanol mixtures at various temperatures.* Journal of Molecular Liquids, 2017. **241**: p. 1032-1037. 10.1016/j.molliq.2017.06.080.
14. Li, W., et al., *Solubility measurement, correlation and mixing thermodynamics properties of dapsone in twelve mono solvents.* Journal of Molecular Liquids, 2019. **280**: p. 175-181. 10.1016/j.molliq.2019.02.023.
15. Li, X., et al., *Solubility of hydroquinone in different solvents from 276.65 K to 345.10 K.* Journal of Chemical and Engineering Data, 2006. **51**(1): p. 127-129. 10.1021/je0502748.
16. Heryanto, R., M. Hasan, and E.C. Abdullah, *Solubility of isoniazid in various organic solvents from (301 to 313) K.* Journal of Chemical and Engineering Data, 2008. **53**(8): p. 1962-1964. 10.1021/je800156m.

17. Yuan, Y., et al., *Solubility Determination and Modeling of p-Nitrobenzamide Dissolved in Twelve Neat Solvents from 283.15 to 328.15 K.* Journal of Chemical and Engineering Data, 2019. **64**(4): p. 1840-1850. 10.1021/acs.jced.9b00065.
18. Shi, J., et al., *Solubility Measurement and Correlation of Probenecid in 12 Pure Organic Solvents and Thermodynamic Properties of Mixing of Solutions.* Journal of Chemical and Engineering Data, 2019. **64**(2): p. 624-631. 10.1021/acs.jced.8b00863.
19. Nordström, F.L. and Å.C. Rasmuson, *Solubility and melting properties of salicylic acid.* Journal of Chemical and Engineering Data, 2006. **51**(5): p. 1668-1671. 10.1021/je060134d.
20. Romdhani, A., et al., *Solubility of sulfacetamide in (ethanol + water) mixtures: Measurement, correlation, thermodynamics, preferential solvation and volumetric contribution at saturation.* Journal of Molecular Liquids, 2019. **290**. 10.1016/j.molliq.2019.111219.
21. Qu, H., et al., *Role of solvent properties and composition on the solid-liquid equilibrium of trifloxystrobin and thermodynamic analysis.* Journal of Molecular Liquids, 2019. **294**. 10.1016/j.molliq.2019.111566.
22. Yu, Z., et al., *Measurement and Correlation of Solubility and Thermodynamic Properties of Vinpocetine in Nine Pure Solvents and (Ethanol + Water) Binary Solvent.* Journal of Chemical and Engineering Data, 2019. **64**(1): p. 150-160. 10.1021/acs.jced.8b00663.
23. Delgado, D.R., et al., *Solution thermodynamics and preferential solvation of sulfamethazine in (methanol + water) mixtures.* Journal of Chemical Thermodynamics, 2016. **97**: p. 264-276. 10.1016/j.jct.2016.02.002.
24. Delgado, D.R. and F. Martínez, *Solubility and solution thermodynamics of sulfamerazine and sulfamethazine in some ethanol+water mixtures.* Fluid Phase Equilibria, 2013. **360**: p. 88-96. 10.1016/j.fluid.2013.09.018.
